# Anesthesiologists’ Assessment of the Correctness, Completeness, and Coherence of ChatGPT Answers to Patient Preoperative Questions

**DOI:** 10.7759/cureus.112602

**Published:** 2026-07-13

**Authors:** Daniel Werry, Garrett Barry, Vishal Uppal, Jonathan G Bailey

**Affiliations:** 1 Department of Anesthesiology, Pharmacology and Therapeutics, University of British Columbia, Vancouver, CAN; 2 Department of Anesthesiology, Pharmacology and Therapeutics, University of British Columbia, Victoria, CAN; 3 Department of Anesthesia, Pain Management &amp; Perioperative Medicine, Dalhousie University, Halifax, CAN

**Keywords:** anesthesiology, artificial intelligence, chatgpt, patient questions, preoperative

## Abstract

Introduction

Patients are increasingly turning to large language models (LLMs) for answers. However, LLMs like ChatGPT are relatively new technology and are untested for this purpose. Inconsistency and “hallucinations” are commonly described problems with LLMs. This study tested the consistency, correctness, completeness, and coherence of answers to frequently asked patient questions, as scored by expert anesthesiologists.

Methods

We gathered commonly asked patient questions from professional association websites. Patient partners were consulted to confirm relevance of the questions. A subset of questions was tested for consistency using intraclass correlation ICC (3,k) across multiple leading prompts. Then anesthesiologists and trainees scored the responses on a Likert scale for 10 anesthesia-related patient questions, with and without a leading prompt. Interrater agreement was calculated, and Likert scores were described.

Results

In terms of consistency, all ICC (3,k) values were below the prespecified threshold of 0.75, although confidence intervals were wide. Overall median ratings of the correctness, completeness, and coherence of each question ranged from 3.4 to 3.9, corresponding to average ratings between “good” and “very good.” Ratings for all responses were similar regardless of whether the question was submitted with or without the prompt. Excellent scores were rarer, receiving 81 (16%) for correctness, 66 (13%) for completeness, and 121 (23%) for coherence.

Discussion

ChatGPT offers appropriate and moderately consistent answers to anesthesia-related patient questions. Anesthesiologists should be aware that while ChatGPT responses are generally correct, complete, and coherent, this does not translate to patient comprehension, and the answers may not be appropriate for the particular patient and institution.

## Introduction

The availability and use of large language models (LLMs) has rapidly expanded over the last few years. This rapid growth has seen many new use cases, including for medical information. Patients may not have an opportunity to speak to an anesthesiologist before the day of their surgery and instead turn to alternate sources of information. LLMs such as Open AI’s Chat Generative Pre-trained Transformer (ChatGPT) may be preferred resources for patients to have medical questions answered in the absence of a healthcare professional. Indeed, ChatGPT responses to patient questions can be higher quality and more empathetic than responses from actual physicians [1].

Limitations of ChatGPT include incorrect or missing citation of source material [2], poor readability [3], diminished performance answering questions requiring nuanced decision-making [4], dependence on appropriate prompting from users [5], and potential to fabricate or “hallucinate” (confidently give incorrect or misleading responses) [6]. It is important that anesthesiologists are aware of the platforms’ limitations to ensure their patients are informed.

Research to date on ChatGPT responses to patient questions about their upcoming anesthetic has focused on accuracy and reading level [3,7-9], but less is known about consistency (whether answers change with repeated or different prompts) and coherence (logic and flow) of answers, which may influence patient understanding and thus preparedness to engage in shared decision-making about their anesthetic care.

The purpose of this study was to evaluate anesthesiologists’ impression of the consistency, correctness, completeness, and coherence of GPT-4 responses to patient questions. We hypothesized that the responses would not be consistent but would be largely correct, complete, and coherent. The survey results will provide a better understanding of the current limitations and potential for ChatGPT to aid in educating patients on common anesthetic-related questions. The primary outcome was to define the degree of correctness. Secondarily, we sought to define the degree of completeness, and coherence. Prior to asking experts to score correctness, completeness, and coherence, it was necessary to define consistency across various prompts and repeated questions.

## Materials and methods

Design

We obtained ethical approval from the Nova Scotia Health Research Ethics Board (REB# 1029682) for this prospective observational study. This study was planned in two phases. The first phase of this study was to establish the level of consistency between answers generated by ChatGPT 4.0 (OpenAI, San Francisco, CA, USA). Once consistency of responses was established, the second phase was a survey of anesthesiologists across multiple subspecialties who rated the correctness, completeness, and coherence of responses to patient questions.

ChatGPT-4 use

The investigators used the publicly available paid web application of ChatGPT-4-turbo in March and April 2024. The default parameters were used to best represent typical use by the public. At the time the study was carried out, the parameter defaults of the ChatGPT website were not publicly disclosed by OpenAI. The application programming interface documentation provides parameter definitions but did not specify default values for the web‑app. The estimated default parameters, such as temperature, top_p, and penalties at the time of the study, as indicated by ChatGPT-4, are given in Appendix A, which are based on the archived OpenAI documentation. Temperature relates to the randomness of the responses generated, with low values indicating more factual and precise answers, while higher values indicate more creativity and divergence. Top_p is another indicator of randomness by controlling the pool of words used to generate answers, with low values indicating concise, deterministic answers, while high values indicate broader answers with increased vocabulary. Penalties suppress repetitiveness such that higher values will use more varied language.

Anesthesia-related patient questions

We gathered commonly asked patient questions from the webpages of the Royal College of Anaesthetists [10], the Australian Society of Anaesthetists [11], and the Canadian Anesthesiology Society [12], and redundant questions were removed, yielding a list of 17 questions. Those questions were then reviewed by a team of patient partners to select the most pertinent questions. The patient partner group (several former patients who have been trained to give lay feedback on studies) recommended reducing the total number of questions to 10 to reduce survey length and selected the questions they believed to be the most useful to patients in a perioperative setting (Table 1).

**Table 1:** Frequently asked anesthesia-related patient questions gathered from professional association websites. Source: [10-12]

Frequently asked anesthesia-related patient questions
Q1: What happens during a general anesthetic?
Q2: What does regional anesthesia mean and what is a nerve block?
Q3: What are the risks of general anesthesia?
Q4: What are the risks of regional anesthesia?
Q5: Are epidurals dangerous?
Q6: Will the epidural affect my baby?
Q7: Do anesthetic drugs damage the brain?
Q8: Are children more at risk from anesthesia?
Q9: What if I wake up during the operation?
Q10: Why can't I eat or drink before surgery?

Phase 1: consistency

Four of the 10 questions were entered into GPT-4 for consistency testing. One of the investigators queried each question with five unique prompts (Appendix B), and each prompt was submitted four times, for a total of 80 entries. Each entry was submitted in a new chat. All ChatGPT responses were recorded into a single document for scoring by investigators. A subset of the questions was scored for consistency for pragmatic reasons, given that this produced an adequate number of ratings to quantitatively judge consistency and given that the same prompts were used across all questions.

Two investigators reviewed each of the GPT-4 answers. Scoring rubrics were created a priori based on the answers supplied on the anesthesia society webpages (Appendix C). These acted as the “standard-of-care" answers taken from four national professional anesthesiology associations from countries with well-developed healthcare systems. Answers were broken down into component parts, and investigators assigned a score of inadequate = 0, partial = 1, or complete = 2 for each answer component. Two investigators came to consensus on each of the scores, and where no consensus was met, a third investigator resolved the disagreement. To examine the consistency of GPT-4’s answers to a single question with varying prompts, intraclass correlation (ICC (3,k)) analysis was applied to the 20 answers for each of the four questions [13].

Prior studies have defined ICC (3,k) values of greater than 0.75 indicative of good reliability [13]. A priori, it was decided that if the ICC (3,k) was less than 0.75 between answers, each question for the survey (Phase 2) would be repeated with and without a leading prompt to account for variability in answers during the second phase of the study.

Phase 2: correctness, completeness, and coherence

The questions, along with the corresponding GPT-4-generated responses, were built into a Research Electronic Data Capture (REDCap) survey to be assessed and rated by practicing anesthesiologists (Appendix D). Answer consistency with different prompts did not meet our prespecified threshold, and thus each question was submitted for the survey with and without the prompt, “I am a patient that is about to have an operation. Please answer my questions about anesthesia,” to characterize the impact of a realistic prompt on perceived answer quality. The prompt was chosen based on investigator consensus as to what a typical patient may enter into ChatGPT.

Survey respondents rated each GPT-4 generated answer using the following three criteria:

· Correctness: the information in the answer is accurate and up to date

· Completeness: the answer provides all the relevant information required to address the question

· Coherence: the answer makes sense and flows well, grammatically, and in terms of meaning

Each criterion was assessed for each artificial intelligence (AI)-generated response using a 5-point Likert scale (1=poor, 2=somewhat good, 3=good, 4=very good, 5=excellent).

Survey participants

Survey participants were recruited from anesthesiologists (including staff, fellows, and trainees) at a single academic center in Halifax, Canada (Dalhousie University Department of Anesthesia, Pain Management & Perioperative Medicine), which includes subspecialty areas such as obstetric anesthesia, pediatric anesthesia, cardiac anesthesia, thoracic anesthesia, and regional anesthesia. Survey invitations were emailed to all practicing anesthesiologists in the department to maximize diversity. Resident anesthesiologists were included as well, but participation was limited to five residents so as not to skew results towards inexperienced practitioners. Nevertheless, patients are likely to interact with and ask these questions of trainees during their anesthetic care, and thus trainees represent a likely real-life alternative to asking these questions of ChatGPT. Participation was voluntary with a $50 incentive for fully completed surveys. Demographics of survey respondents were captured with survey questions.

Statistical analysis

A data analysis and statistical plan was written and filed with the research ethics board before data were accessed. The analysis was updated in response to ongoing statistical consulting after the data became available. The correctness, completeness, and coherence of each question is reported by median and interquartile Likert scale scores. The interrater agreement was calculated using the intraclass correlation coefficient, which informs if median ratings are representative of overall rather than divergent opinion. ICC (3,k) was chosen over Cohen’s kappa as the latter may be biased in cases where the categories are imbalanced [14]. In this case, the intraclass correlation indicates agreement between expert raters rather than the consistency of ChatGPT-4 responses.

Sample size calculation

The initial aim of the study was descriptive, and, as such, the precision of the estimate in Likert scales was calculated to determine an acceptable sample size. Assuming a standard deviation of 1.25 for responses on a Likert scale of 1-5, a sample size of 24 participants would allow us to estimate the population mean with a 95% confidence interval that is no wider than ±0.5, with 80% power.

## Results

Surveys rating the correctness, completeness, and coherence of GPT-4 responses to 10 patient pre-anesthetic questions were sent to 147 anesthesia faculty and residents. Of the 147 recipients of the survey, 50 participants opened the survey and 26 completed it (Table 2). Several anesthetic subspecialties are represented, and the majority of participants have been in independent practice for more than 10 years.

**Table 2:** Demographics of respondents scoring ChatGPT responses to frequently asked patient questions.

Demographics	Respondents
Age (years), n/N (%)	
20-30	2/26 (8%)
31-40	7/26 (27%)
41-50	8/26 (30%)
51-60	7/26 (27%)
>60	2/26 (8%)
Female, n/N (%)	9/26 (35%)
Experience (years), n/N (%)	
Trainee	4/26 (15%)
1-5	4/26 (15%)
6-10	4/26 (15%)
11-20	7/26 (27%)
>20	7/26 (27%)
Specialty, n/N (%)	
Cardiac anesthesia	1/26 (4%)
Regional or pain anesthesia	5/26 (19%)
Thoracic, airway, major abdominal anesthesia	5/26 (19%)
Obstetric anesthesia	3/26 (12%)
Pediatric anesthesia	4/26 (15%)
Other or undefined	8/26 (31%)

Prior to survey distribution, the consistency of GPT-4 responses to a subset of questions submitted repeatedly and with varying prompts was tested (Appendix B). Investigators rated the responses according to a scoring rubric (Appendix C), and consistency was judged by intraclass correlation (ICC (3,k)) of response ratings. All ICC (3,k) values were below the prespecified threshold of 0.75, although confidence intervals were wide (Table 3). Thus, each of the 10 survey questions was submitted with and without a leading prompt to examine whether a single prompt may impact answer quality, in addition to consistency, as judged in the surveys. Unfortunately, this study did not have the statistical power to examine the effect of each type of variation separately to understand whether repeat entry or prompt variation played a larger role.

**Table 3:** Consistency of GPT-4 responses to a subset of pre-anesthetic patient questions

Question	ICC (3,k) [95% CI]
1. What happens during a general anesthetic?	0.65 [0.34–0.96]
2. What are the risks of general anesthesia?	0.64 [0.36–0.94]
3. What does regional anesthesia mean and what is a nerve block?	0.58 [0.33–0.90]
4. What are the risks of regional anesthesia?	0.42 [0.22–0.80]

**Figure 1:**
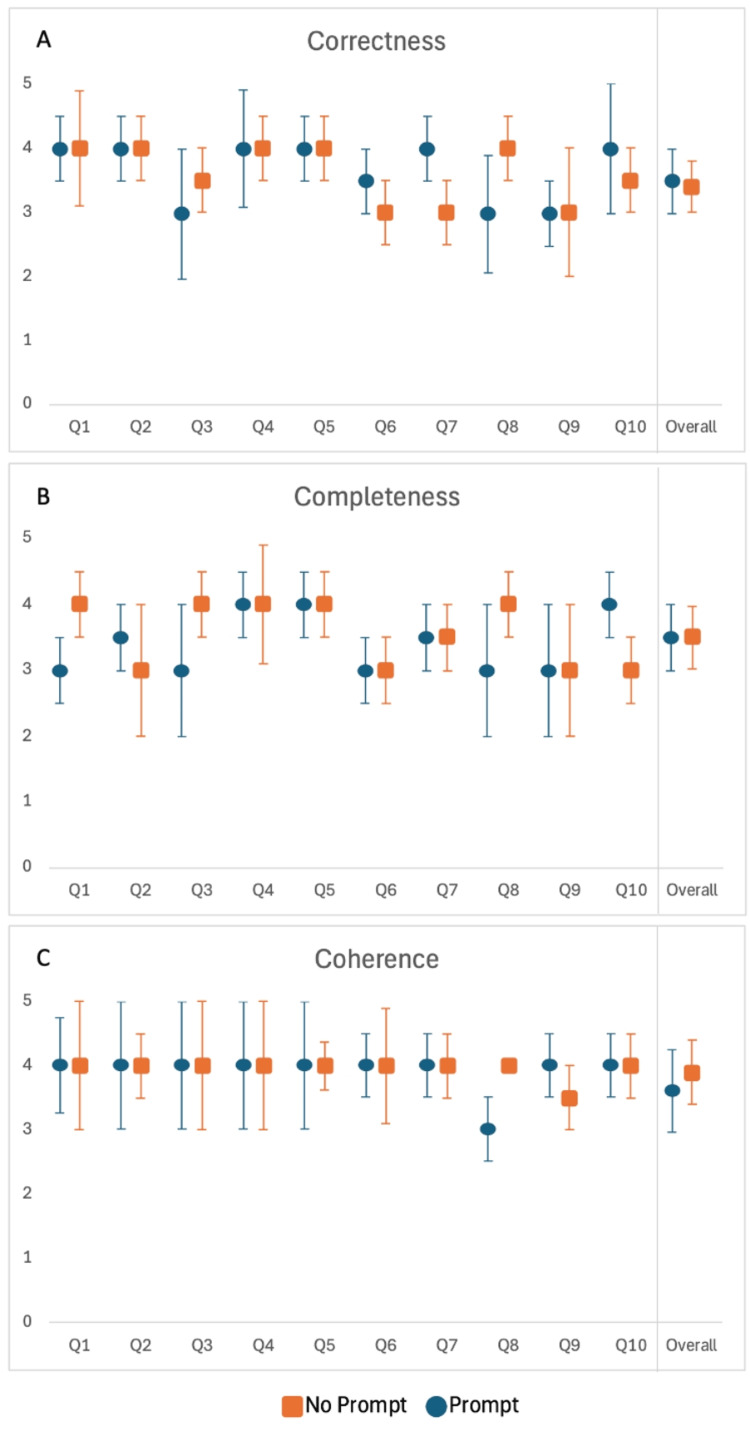
Correctness, completeness, and coherence of ChatGPT answers to common anesthesia-related patient questions with and without a leading prompt. Each graph displays either (A) correctness, (B) completeness, or (C) coherence across each of the 10 questions and one overall average rating for each graph. Since each question was entered into ChatGPT with and without a prompt, two ratings are presented for each question. Squares = no prompt; circles = prompt

Anesthesia practitioners rated the correctness, completeness, and coherence of each GPT-4 response according to a 5-point Likert scale. Overall median ratings of the correctness, completeness, and coherence of each question ranged from 3.4 to 3.9 (Table 4), corresponding to average ratings between “good” and “very good.” Ratings for all responses were similar regardless of whether the question was submitted with or without the prompt (Figure 1; Table 5). Overall, median [Q1 to Q3] correctness was scored as 3.4 [3.1 to 4.0] without a prompt and 3.5 [2.8 to 3.8] with a prompt (Table 4). In terms of correctness, 138 (27%) responses were scored as “good” and 205 (39%) as “very good” (Appendix E). Completeness was scored as 3.5 [2.9 to 3.9] without a prompt and 3.5 [2.7 to 3.7] with a prompt. In total, 146 (28%) responses were scored “good” and 191 (37%) as “very good” (Appendix F). ChatGPT received coherence scores of 3.9 [3.3 to 4.4] without prompting and 3.6 [3.1 to 4.4] with prompting. Considering categories, 132 (25%) responses were scored as “good,” while 199 (38%) were scored “very good” (Appendix G). Excellent scores were rarer, receiving 81 (16%) for correctness, 66 (13%) for completeness, and 121 (23%) for coherence.

**Table 4:** Correctness, completeness, and coherence of ChatGPT answers to common anesthesia-related patient questions with and without a leading prompt. Note: N = 26, no missing data.

Question	Correctness, median [IQR]		Completeness, median [IQR]		Coherence, median [IQR]	
	No prompt	Prompt	No prompt	Prompt	No prompt	Prompt
Q1: What happens during a general anesthetic?	4 [3.0, 4.8]	4 [3.0, 4.0]	4 [3.0, 4.0]	3 [3.0, 4.0]	4 [3.0, 5.0]	4 [3.2, 4.8]
Q2: What does regional anesthesia mean and what is a nerve block?	4 [3.0, 4.0]	4 [3.0, 4.0]	3 [2.0, 4.0]	3.5 [3.0, 4.0]	4 [3.0, 4.0]	4 [3.0, 5.0]
Q3: What are the risks of general anesthesia?	3.5 [3.0, 4.0]	3 [2.0, 4.0]	4 [3.0, 4.0]	3 [2.0, 4.0]	4 [3.0, 5.0]	4 [3.0, 5.0]
Q4: What are the risks of regional anesthesia?	4 [3.0, 4.0]	4 [3.0, 4.8]	4 [3.0, 4.8]	4 [3.0, 4.0]	4 [3.0, 5.0]	4 [3.0, 5.0]
Q5: Are epidurals dangerous?	4 [3.0, 4.0]	4 [3.0, 4.0]	4 [3.0, 4.0]	4 [3.0, 4.0]	4 [4.0, 4.8]	4 [3.0, 5.0]
Q6: Will the epidural affect my baby?	3 [3.0, 4.0]	3.5 [3.0, 4.0]	3 [3.0, 4.0]	3 [3.0, 4.0]	4 [3.0, 4.8]	4 [3.0, 4.0]
Q7: Do anesthetic drugs damage the brain?	3 [3.0, 4.0]	4 [3.0, 4.0]	3.5 [3.0, 4.0]	3.5 [3.0, 4.0]	4 [3.0, 4.0]	4 [3.0, 4.0]
Q8: Are children more at risk from anesthesia?	4 [3.0, 4.0]	3 [2.2, 4.0]	4 [3.0, 4.0]	3 [2.0, 4.0]	4 [4.0, 4.0]	3 [3.0, 4.0]
Q9: What if I wake up during the operation?	3 [2.0, 4.0]	3 [3.0, 4.0]	3 [2.0, 4.0]	3 [2.0, 4.0]	3.5 [3.0, 4.0]	4 [3.0, 4.0]
Q10: Why can't I eat or drink before surgery?	3.5 [3.0, 4.0]	4 [2.0, 4.0]	3 [3.0, 4.0]	4 [3.0, 4.0]	4 [3.0, 4.0]	4 [3.0, 4.0]
Total average score (average of Q1 to Q10)	3.4 [3.1, 4.0]	3.5 [2.8, 3.8]	3.5 [2.9, 3.9]	3.5 [2.7, 3.7]	3.9 [3.3, 4.4]	3.6 [3.1, 4.4]

Intraclass correlation was used to assess whether ratings received a broad consensus or were the result of divergent opinion among the expert raters. Overall ICC (3,k) values were between 0.5 and 0.7 for each of correctness, completeness, and coherence, suggesting moderate agreement between anesthesiologists, albeit with low confidence (Table 5). Agreement was similar regardless of whether the questions were submitted with or without a prompt. Evidence of divergent opinion could be seen in select questions where a minority of anesthesiologists rated responses as poor. For example, 3/26 anesthesiologists judged the correctness of responses to “what if I wake up?” and “why can’t I eat or drink?” as poor, and 4/26 anesthesiologists judged completeness to be poor for the response to “are children at more risk of anesthesia?” (Appendix C). When judging coherence, there were no questions with more than two poor ratings.

**Table 5:** Intraclass correlations of the correctness, completeness, and consistency indicating agreement between experts rating ChatGPT-4 responses. Note: N = 26, no missing data.

	ICC (3,k)
ChatGPT receives no prompt	
Correctness	0.65 [0.24, 0.90]
Completeness	0.65 [0.24, 0.90]
Coherence	0.41 [-0.28, 0.83]
ChatGPT is prompted	
Correctness	0.63 [0.20, 0.89]
Completeness	0.64 [0.21, 0.89]
Coherence	0.69 [0.33, 0.91]
Prompt and no prompt conditions combined	
Correctness	0.64 [0.37, 0.83]
Completeness	0.66 [0.40, 0.84]
Coherence	0.59 [0.27, 0.81]

## Discussion

This study evaluated anesthesiologists’ impressions of the correctness, completeness, and coherence of GPT-4 responses to commonly asked patient questions about anesthesia. We found that across all domains, on average, anesthesiologists rated the GPT-4 responses to patient questions as "good" to "very good." However, consistency analysis showed evidence of divergent opinion. On select questions, a minority of anesthesiologists rated the correctness and completeness of responses as poor. Coherence was generally rated as "good" to "very good," with "poor" ratings being rare.

The central question for health systems and decision-makers is whether LLMs, such as ChatGPT, provide good quality information on a consistent basis. A recent systematic review found that LLMs are being used for a wide variety of tasks in anesthesiology, including generating risk prediction scores, informed consent documents, clinical notes, predicting clinical outcomes, outlining assessment and treatment recommendations, and designing educational patient handouts [9]. False, variable, incomplete, or incoherent information in high-stakes medical subspecialties such as anesthesiology is of particular concern for patient safety and provider liability. Therefore, before LLMs are endorsed as patient resources for anesthesia, how closely the advice they give approximates advice from expert anesthesiologists must be rigorously examined. This was the objective of the present study.

Separate from the survey, we analyzed the consistency of GPT-4 responses to a subset of patient questions when repeated with different prompts. Consistency ratings combine two different types of consistency: (1) whether it was two different patients entering a similar prompt (repeat entry) and (2) whether it was two different patients using dissimilar prompts (prompt variation). Responses were only moderately consistent, and therefore answers from GPT-4 may vary with different prompts. These findings imply that while GPT-4 answers may vary with different prompts, the overall correctness, completeness, and coherence did not vary, at least for the prompt that was used. This aligns with previous research evaluating various ChatGPT models to provide answers based on osteoarthritis evidence-based guidelines [15]. ChatGPT-4-web showed the strongest consistency of the model tests, with scores between 50.6% and 63%. Interestingly, GPT-3.5-ft-0 and GPT-3.5-API-0 demonstrated near-perfect reliability across prompts, while other models showed only moderate or lower agreement [15]. Conversely, LLMs answering questions on a dietitian exam had consistency (as measured by Cohen’s Kappa) much higher than our results [16]. The lowest coefficient was 0.902, with ChatGPT-4o being the most consistent across responses. It also maintained high consistency in answering the same questions multiple times [16]. This variability is similar to what might be expected from anesthesiologists, who may exhibit behaviors analogous to prompt variation (responses vary depending on patient differences) and repeat entry (responses vary between anesthesiologists). However, regardless of the source (human or LLM), patient information is expected to meet professional standards of quality and accuracy despite this variability in responses.

There are several factors that may impact the quality of LLM responses. Incorrect responses by LLMs may be caused by “AI hallucination,” probably better thought of as confabulations [17]. Therefore, LLMs may provide inaccurate answers on nuanced topics with varying opinions [18,19] or on complex material with limited available literature [20]. Nevertheless, specific to frequently asked patient questions about anesthesia, prior studies have found ChatGPT responses to be reasonable [8] and accurate [7] and, for confabulation, to be rare compared to other LLMs [3]. Our findings are similar to other areas of medicine. Four LLMs (ChatGPT-40, Claude 3.5 Sonnet, Gemini Advanced, and Llama 3.1 405B) outperformed human physicians at identifying rare diseases, with Claude demonstrating the highest performance [21-24]. While these findings are impressive, there remains inaccuracies, suggesting that these models may not be appropriate for high consequence questions. Specific to regional anesthesia, a pilot study found that when faced with clinical scenarios, LLMs demonstrated weaknesses in clinical judgement and rationale for their suggested course of action [18]. Furthermore, there were safety concerns with their suggested actions in some cases, and they often did not explain when certain blocks would be inappropriate, displaying concerns for their use as clinical decision aids [18]. Overall, the accuracy of responses seems to be adequate as a patient resource, especially when paired with expert consultation. This may reduce patient anxiety, address concerns, and improve efficiency of preoperative patient discussions. This could be particularly useful in settings where access to expert consultation is restricted due to workload and understaffing.

This study adds to the growing body of literature on LLM use in pre-anesthetic patient education in several ways. The majority of previous studies examining the ability of ChatGPT to answer patient questions have focused on GPT-3.5, instead of the more recent and capable GPT-4 model [9]. In contrast to accuracy and completeness, the coherence of responses has rarely been examined. One recent study rated the “understandability” of GPT-4 answers to commonly asked anesthetic questions highly, similar to our study’s findings for coherence [25]. However, GPT-4 performed poorly in accuracy and completeness of responses [25]. One possible explanation for poor ratings in medical content performance could relate to responses that are divergent between medical literature and institutional guidelines but may be otherwise correct, such as the difference in fasting guidelines. Finally, our study uniquely reports the impact of question prompts on the consistency of responses to pre-anesthetic questions. Patients are likely to use varied prompts, and it is important to evaluate how this may impact response quality. In our study, while different prompts did impact GPT-4 responses, when a single patient-centered prompt was used, the quality of answers was unchanged. It is possible that a different prompt for the survey would have yielded different results.

There are some potential limitations of this study. Our response rate was low at 34% (50/147), and of those that opened the survey, only 52% completed it (26/50). The overall completion rate of 18% (26/147) may bias results towards people interested in the use of LLMs. However, we did achieve broad representation across anesthetic subspecialties and experience levels, which is a strength of the study. The sample size calculation was based on precision of point estimates rather than ICC calculation since the statistical plan changed after initiating the study. The ICC estimates are somewhat underpowered, which resulted in wider confidence interval than is optimal. Our study only investigates common wording used in major anesthesiology associations, and we were limited by survey length to the inclusion of only one additional prompt. Anesthesiologists may not be the best judge of what is coherent to a patient, and the readability of ChatGPT, previously described as college-level [3], may compromise patient comprehension. Nonetheless, coherence is an important measure, and readability can be adjusted with prompts. We used subjective expert ratings rather than objective measures of factual accuracy or patient outcomes; however, there is some inherent subjectivity when considering an adequate response to a patient question and allowing many types of responses. In this study, the survey respondents knew that they were evaluating AI-generated responses. It is possible that if they viewed generative AI negatively, particularly when evaluating whether it could provide information comparable to an anesthesiologist, they would be more prone to rate the responses negatively. Future studies may consider blinding or intentional deception to conceal AI-generated responses. Additionally, only one generative AI was tested in this study. LLMs vary in their ability to answer complex medical questions [26,27]. We focused on a single LLM in order to maintain completion rates while allowing for several aspects of performance to be evaluated in a rigorous manner. Finally, although we used the most recent model of ChatGPT available at the time of the survey, this assessment is only a snapshot in time as these models have continued to evolve. It is possible that over time as ChatGPT undergoes further refinement, the answers to common questions could change in their correctness, completeness, and coherence.

## Conclusions

ChatGPT offers appropriate and moderately consistent answers to anesthesia-related patient questions. Anesthesiologists should be aware that, while in our study, ChatGPT responses were considered to be correct, complete, and coherent, this does not translate to patient comprehension, and the answers may not be appropriate for the particular patient and institution. Variability in responses is expected and dependent on the LLM used, prompts entered, and chat history. Further discussion between the patient and physician is required to clarify questions and ensure informed care. Future research should assess consistency between different prompt strategies versus reliability of answers across repeated questions. Research on consistency, correctness, completeness, and coherence will need to match the pace of LLM development as new models are introduced.

## Appendices

Appendix A

**Table 6:** Parameters of ChatGPT-4 found in archived OpenAI documentation from March 2024.

Parameter	Default Value
temperature	1
top_p	1
frequency_penalty	0
presence_penalty	0
max_tokens	Not set by default (user-defined)
stop	null (i.e., no stop sequence)
model	gpt-4 (or gpt-4-turbo)

Appendix B

Subset of frequently asked questions with varying prompts to establish consistency in answers.

Questions

1. What happens during a general anesthetic?

2. What are the risks of general anesthesia?

3. What does regional anesthesia mean and what is a nerve block?

4. What are the risks of regional anesthesia?

Prompts

1. Act as an expert anesthesiologist who is providing answers to a curious patient.

2. Act as an anesthesiologist who is providing answers to an anxious patient.

3. I’m a patient that is going to have an operation. Please answer my questions about anesthesia.

4. [no initial prompt]

5. Act as a devil’s advocate [inviting a contrary or opposing opinion to conventional responses].

Appendix C

**Table 7:** Scoring rubric for subset of frequently asked questions about anesthesia by patients using standard-of-care answer elements from society websites. PDPH, postdural puncture headache

	Inadequate = 0	Partial = 1	Good = 2
What happens during a general anesthetic?			
Standard-of-care answer elements			
You are unconsciousness and feel no pain during surgery			
Performed by highly trained doctors and providers			
Medications or gas given			
Essential for some operations, there may be other options (e.g. regional anesthesia)			
What are the risks of general anesthesia?			
Standard-of-care answer elements			
The risks are very low for healthy people			
Risk depends on your health, surgical procedure, and anesthetic technique			
Awareness, death, vision loss (very rare)			
Nausea, sore throat, thirst, shivering, pain at injection site, minor lip/tongue injury (common)			
Peripheral nerve injury, dental damage, corneal abrasion, anaphylaxis (rare)			
What does regional anesthesia mean and what is a nerve block?			
Standard-of-care answer elements			
Numb an area of the body so you don’t feel pain			
May also receive sedation			
Local anesthesia is injected around nerves			
May be safer than general anesthesia for some people			
Helps with pain after surgery			
Anesthesia provider uses ultrasound guidance to see the nerves			
What are the risks of regional anesthesia?			
Standard-of-care answer elements			
The risks are generally very low			
Nerve injury (persistent motor/sensory changes)			
Bleeding/hematoma			
Infection			
Pneumothorax / diaphragmatic paresis / dyspnea			
Headache (PDPH)			
Local anesthesia systemic toxicity (LAST)			

Appendix D

ChatGPT-4 responses to frequently asked patient questions as presented to expert reviewers to score correctness, completeness, and coherence.

Phase 2 questions

What happens during a general anesthetic?

What does regional anesthesia mean and what is a nerve block?

What are the risks of general anesthesia?

What are the risks of regional anesthesia?

Are epidurals dangerous?

Will the epidural affect my baby?

Do anesthetic drugs damage the brain?

Are children more at risk from anesthesia?

What if I wake up during the operation?

Why can’t I eat or drink before surgery?

Prompts

No initial prompt

I’m a patient that is going to have an operation. Please answer my questions about anesthesia.

1.

a. [no prompt] What happens during a general anesthetic?

During a general anesthetic, the process typically involves several stages designed to ensure the patient is safely and effectively put to sleep, rendering them unconscious and free of pain for the duration of a surgical procedure. Here’s a breakdown of what happens:

1. **Preoperative Assessment**: Before undergoing anesthesia, patients usually undergo a thorough medical evaluation. This includes reviewing their medical history, medications, allergies, and any previous reactions to anesthesia. The anesthesiologist might also discuss the plan for anesthesia and post-operative pain management.

2. **Preparation**: On the day of the surgery, the patient may be given premedication, which can help relax them and may include medications to reduce the risk of nausea and other side effects.

3. **Induction**: The actual process of entering anesthesia begins with induction, which is often achieved through intravenous (IV) drugs. Sometimes, a gas that is inhaled may also be used. The anesthesiologist administers these drugs to induce a state of unconsciousness. During this phase, the patient rapidly falls asleep.

4. **Maintenance**: Once the patient is unconscious, the anesthesiologist uses a combination of inhaled and intravenous medications to maintain anesthesia. The depth of anesthesia is carefully controlled and monitored. Vital signs such as heart rate, blood pressure, oxygen levels, and breathing are continuously monitored to ensure the patient remains stable.

5. **Intubation and Ventilation**: For most major surgeries, the patient's airway is secured using a tube inserted into the trachea (intubation). This helps maintain proper breathing during the procedure. The patient may be connected to a mechanical ventilator which takes over the breathing process.

6. **Monitoring**: Throughout the procedure, specialized equipment is used to monitor the patient’s critical functions. This includes ECG (heart activity), blood pressure monitor, pulse oximetry (oxygen levels in the blood), and capnography (CO2 levels). The anesthesiologist adjusts the anesthetic delivery in response to these readings.

7. **Recovery**: After the surgery is complete, the anesthesiologist reverses the effects of the anesthesia. This involves discontinuing the anesthetic drugs and ensuring that the patient begins to regain consciousness and resume breathing on their own. The patient is then moved to a recovery area where they continue to be monitored until they wake up fully and the immediate effects of the anesthesia have worn off.

8. **Post-Operative Care**: Post-anesthesia care includes managing any pain or side effects from the surgery and anesthesia. Nurses and doctors monitor the patient’s recovery and provide necessary medications to manage pain, nausea, or other symptoms.

General anesthesia is a critical component of modern surgery, enabling complex operations to be performed without discomfort or pain for the patient. It requires careful planning, precise dose adjustment, and continuous monitoring to ensure safety and effectiveness.

b. [I’m a patient that is going to have an operation. Please answer my questions about anesthesia] What happens during a general anesthetic?

During a general anesthetic, you will be put into a state of controlled unconsciousness, effectively ensuring that you do not feel pain or have any awareness during the surgery. Here’s what generally happens:

1. **Pre-Operation Preparation**: Before the operation, you’ll meet with an anesthesiologist to discuss your medical history, any allergies you may have, and your previous experiences with anesthesia, if any. This is to ensure that the anesthesia plan is tailored specifically to your needs.

2. **Induction**: When it's time for your surgery, an anesthesiologist will administer the general anesthetic. This is usually done through an intravenous (IV) line in your arm, but it can also involve breathing in anesthetic gases through a mask. You will gradually lose consciousness.

3. **Maintenance**: Once you are unconscious, the anesthesiologist will continue to deliver the anesthesia (either through the IV or through breathing gases) to keep you in a state of unconsciousness throughout the surgery. During this phase, your breathing, heart rate, blood pressure, and other vital signs are closely monitored and maintained at safe levels. The anesthesiologist adjusts the levels of anesthesia as needed based on these vital signs and the progress of the surgery.

4. **Monitoring**: Throughout the procedure, the anesthesiologist monitors your condition and ensures that you stay asleep and pain-free. Modern monitoring equipment is used to continuously check your heart rate, blood pressure, oxygen levels, and other critical parameters.

5. **Waking Up**: Once the surgery is complete, the anesthesiologist will stop the administration of the anesthetic agents, and you will gradually begin to wake up. This occurs in a recovery room, where your condition is closely monitored to ensure that you wake up safely.

6. **Post-Operation Recovery**: After you wake up, you may feel groggy or disoriented for a short time. Some people experience side effects like nausea, dry mouth, or chills, but these generally subside relatively quickly. Nurses in the recovery room will continue to monitor your vital signs and provide pain relief or other necessary medications as you recover.

The process is designed to make sure you are safe and pain-free throughout your surgical experience.

2.

a. What does regional anesthesia mean and what is a nerve block?

Regional anesthesia is a type of anesthesia that numbs a specific part of the body to prevent any pain in that area during or after a surgical procedure. Unlike general anesthesia, where the patient is unconscious, regional anesthesia allows the patient to remain awake or sedated without affecting their consciousness.

A nerve block is a specific type of regional anesthesia. It involves the injection of an anesthetic near a specific nerve or cluster of nerves to block pain from a specific area of the body. This technique is used to manage pain during and after surgery and can also be used for pain relief in non-surgical situations. There are various types of nerve blocks that can target different areas, such as epidural blocks for the lower body and brachial plexus blocks for the arm, to name just a few.

b. [I’m a patient that is going to have an operation. Please answer my questions about anesthesia]. What does regional anesthesia mean and what is a nerve block?

Regional anesthesia is a type of anesthesia that numbs a specific part of the body to block the sensation of pain, without affecting your consciousness. This technique can be used for surgeries on the limbs, the face, the abdomen, and other areas, depending on the nerve supply to those tissues.

A nerve block is a procedure within the scope of regional anesthesia, where the anesthetic is injected directly around specific nerves. The medication interrupts pain signals traveling along that nerve pathway. This method is often used for procedures on the hands, arms, feet, legs, or face.

There are different types of nerve blocks, such as:

- **Peripheral nerve blocks**, which target the nerves supplying specific areas of the body, like an arm or leg.

- **Plexus blocks**, which target a network of nerves, like the brachial plexus in the shoulder area for arm surgeries.

- **Epidural and spinal blocks**, which are used for lower abdominal, pelvic, rectal, or lower extremity surgery.

Benefits of a nerve block can include less need for general anesthesia, good pain control after surgery, and sometimes a quicker recovery. However, as with any medical procedure, there are risks involved, and it's important to discuss these with your anesthesiologist to understand what's best for your particular situation.

3.

a. What are the risks of general anesthesia?

General anesthesia, which is used during surgery to render a patient unconscious and free from pain, has various potential risks, though serious complications are rare. The risks associated with general anesthesia can vary based on the individual’s health, age, type of surgery, and the duration of the procedure. Here are some common risks:

1. **Allergic Reactions:** Some patients may have allergic reactions to the anesthetics or other medications used, which can range from mild to severe.

2. **Breathing Difficulties:** General anesthesia can affect respiratory function, leading to potential complications such as aspiration (inhaling stomach contents into the lungs), which can cause pneumonia.

3. **Cardiovascular Issues:** Anesthesia can affect heart rate and blood pressure. It might lead to heart attacks or strokes in very rare cases, especially in those with pre-existing cardiovascular conditions.

4. **Nausea and Vomiting:** These are common side effects, especially in the first few hours after surgery, which can be uncomfortable and potentially dangerous if it leads to dehydration or aspiration.

5. **Cognitive Impairment:** Post-operative cognitive dysfunction (temporary confusion, memory loss, and difficulty concentrating) can occur, particularly in older adults. In very rare cases, this can evolve into a more permanent condition.

6. **Nerve Damage:** Depending on the type of surgery and anesthesia, there might be a risk of nerve damage, which can result in numbness or paralysis.

7. **Temperature Regulation Issues:** General anesthesia can impair the body’s ability to regulate temperature, leading to hypothermia.

8. **Malignant Hyperthermia:** A rare but serious genetic reaction to certain anesthetic drugs, causing a rapid rise in body temperature and severe muscle contractions.

The specific risks for any individual undergoing surgery will depend on their particular circumstances, including their overall health and the specifics of the surgery they are undergoing. It's important to discuss all potential risks with your healthcare provider to make informed decisions about surgery and anesthesia.

b. I’m a patient that is going to have an operation. Please answer my questions about anesthesia. What are the risks of general anesthesia?

General anesthesia is a medical procedure used to induce unconsciousness, allowing patients to undergo surgery without pain or awareness. While it is generally safe when administered by an experienced anesthesiologist, there are potential risks associated with general anesthesia, especially in certain populations or those with specific health conditions.

Here are some of the risks and side effects associated with general anesthesia:

1. **Allergic Reactions**: Rarely, patients may have an allergic reaction to the anesthetic agents, which can be severe.

2. **Respiratory Issues**: General anesthesia can affect normal breathing. This is usually well-managed by the anesthesiology team, but problems can occur, especially in those with preexisting respiratory conditions.

3. **Cardiovascular Problems**: There can be effects on heart rate and blood pressure, which are closely monitored during the procedure.

4. **Nausea and Vomiting**: Post-operative nausea and vomiting are common side effects, although medications can be given to minimize these issues.

5. **Post-Operative Confusion or Delirium**: This is more common in older adults and can last for a few days.

6. **Sore Throat**: Due to the placement of the breathing tube during surgery, a sore throat may be experienced after the operation.

7. **Malignant Hyperthermia**: A rare but serious reaction to certain anesthetics that causes a high fever and severe muscle contractions.

8. **Nerve Damage**: Very rarely, positioning or surgical factors can lead to nerve damage.

9. **Anesthesia Awareness**: This is an extremely rare situation where a patient becomes aware during surgery but cannot move or communicate.

10. **Post-Operative Cognitive Dysfunction (POCD)**: Some people, particularly those of advanced age, may experience cognitive dysfunction for a few weeks or months after surgery.

It's important to discuss your individual risk factors with your anesthesiologist before surgery, as they can vary greatly depending on your age, general health, medical history, and the type of surgery you are undergoing. The anesthesiology team will take all necessary precautions to minimize risks before, during, and after your operation.

4.

a. What are the risks of regional anesthesia?

Regional anesthesia is a method used to numb a specific area of the body to prevent pain during and after surgical procedures. While it's generally considered safe and effective, there are several potential risks and side effects associated with its use:

1. **Nerve Damage**: Although rare, there is a risk of nerve damage resulting from needle insertion or the direct effect of the anesthetic on nerves. This can lead to temporary or, in very rare cases, permanent weakness, pain, or numbness.

2. **Infection**: As with any procedure involving a needle, there is a risk of infection at the injection site. Proper sterilization techniques and skin cleaning can minimize this risk.

3. **Bleeding**: A needle can accidentally puncture a blood vessel leading to a hematoma (a collection of blood outside of blood vessels), which might also press on nerves.

4. **Allergic Reactions**: Some people may have allergic reactions to the anesthetic agents used. These reactions can range from mild itching or rash to more severe reactions such as anaphylaxis.

5. **Systemic Toxicity**: If the local anesthetic enters the bloodstream in significant amounts, it can cause systemic toxicity, affecting the heart or brain. Symptoms may include ringing in the ears, a metallic taste in the mouth, tingling around the mouth, dizziness, or convulsions.

6. **Failure of Anesthesia**: Sometimes, regional anesthesia might not adequately block the nerve, resulting in insufficient pain relief.

7. **Hypotension**: Spinal and epidural anesthesia can lead to a drop in blood pressure. This is usually manageable but can be significant in some cases.

8. **Prolonged Numbness or Weakness**: Usually, the effects of the anesthesia wear off after a few hours, but sometimes, numbness or weakness can persist for a longer period.

9. **Headache**: Particularly with spinal and epidural anesthesia, a post-dural puncture headache can occur, which might persist for days or weeks.

10. **Respiratory Depression**: Especially in higher or more extensive nerve blocks, there might be an impact on breathing due to the involvement of muscle groups that aid in respiration.

It's important for patients to discuss their medical history with their healthcare provider, including any previous reactions to anesthetics or other medications, to minimize the risk of complications.

b. I’m a patient that is going to have an operation. Please answer my questions about anesthesia. What are the risks of regional anesthesia?

Regional anesthesia is commonly used for surgeries involving specific areas of the body, such as the limbs, the lower part of the body, or for pain relief after surgery. It involves injecting anesthetic near the nerves to numb the part of the body that requires surgery.

The risks associated with regional anesthesia can include:

1. **Nerve Damage**: While rare, the needle or the catheter used for the anesthetic can damage nerves. This can result in temporary or, very rarely, permanent weakness, pain, or numbness.

2. **Infection**: Any time the skin is broken, there's a risk of infection. However, strict protocols reduce this risk.

3. **Bleeding**: There is a risk of bleeding where the needle is inserted, particularly if the patient has a bleeding disorder or is on blood-thinning medications.

4. **Allergic Reactions**: Some people may have an allergic reaction to the anesthetics used.

5. **Low Blood Pressure**: The anesthetics used can expand blood vessels, leading to a drop in blood pressure.

6. **Difficulty Urinating**: Anesthetics can affect the nerves controlling the bladder, usually temporarily.

7. **Prolonged Numbness or Weakness**: Sometimes, the numbness can extend beyond the expected period but usually wears off eventually.

8. **Epidural Hematoma or Abscess**: If an epidural is used (a type of regional anesthesia), there is a very small risk of a blood clot or abscess forming in the epidural space.

9. **Inadequate Anesthesia**: In some cases, regional anesthesia may not provide complete pain relief, and additional pain control methods may be needed.

10. **Spinal Headache**: If the dura mater (the outer membrane of the spinal cord) is punctured, which can happen particularly with epidural or spinal anesthesia, it can lead to a headache that is typically worse when sitting or standing.

It’s important to discuss your health history, concerns, and any medications you're taking with your anesthesiologist to understand how these risks apply to you and what can be done to minimize them. The specifics can vary depending on the type of regional anesthesia being used (e.g., spinal, epidural, nerve block), your personal health profile, and the context of the surgery. Anesthesia has become very safe, but understanding the potential risks and benefits can help you make an informed decision and prepare for your procedure. Always consult with your healthcare provider for the most personalized and up-to-date information.

5.

a. Are epidurals dangerous?

Epidurals are generally considered to be safe procedures and are commonly used for pain relief during labor, childbirth, and certain types of surgery. However, as with any medical intervention, there are potential risks and side effects associated with epidurals. Some of the side effects and complications that can occur, although they are usually rare, include:

- Low blood pressure, which may cause lightheadedness or nausea.

- Temporary loss of bladder control.

- Itchy skin.

- Nausea or feeling sick.

- Headaches.

- Nerve damage in very rare instances.

The occurrence of serious complications is fortunately low, but they do need to be considered when discussing pain management options. These serious complications can include dural puncture, neurological damage which is usually transient, epidural hematoma, and epidural abscess.

Moreover, some patients may experience a failure to achieve good analgesia, which can sometimes be adjusted for effectiveness. Other complications may include hypotension due to sympatholysis, urinary retention, pruritus (particularly when opioids are used), nausea, altered mental status, and motor blockade, which can lead to an increased risk of falls.

Ultimately, the decision to use an epidural should be based on a careful consideration of the potential benefits and risks, and in consultation with medical professionals. For patients undergoing very painful procedures, the beneficial effects of epidural analgesia often outweigh the associated risks or side effects, especially when alternative pain relief methods are not as effective.

For more detailed information on the risks and side effects of epidurals, you may refer to the resources provided by the NHS and UCSF's Pain Management Education.

b. I’m a patient that is going to have an operation. Please answer my anesthesia-related questions. Are epidurals dangerous?

Epidural anesthesia is a commonly used technique, especially in childbirth and for surgeries involving the lower parts of the body. Like any medical procedure, it has potential risks, but it is generally considered safe when performed by a skilled professional.

Here are some key points about the safety of epidural anesthesia:

1. **Low Risk of Complications:** The overall incidence of serious complications from an epidural is low. Common minor side effects might include localized pain or soreness where the needle is inserted, temporary difficulty urinating, and, in some cases, itchy skin or nausea.

2. **Serious Risks Are Rare:** More serious risks associated with epidurals include infection, bleeding, or nerve damage, but these are extremely rare. The risk of permanent harm is very low.

3. **Monitoring and Management:** During the procedure, your vital signs and the drug's effects are closely monitored by an anesthesiologist or a nurse anesthetist. This careful monitoring helps prevent complications.

4. **Individual Suitability:** Not everyone is a candidate for an epidural, and your medical history, current health status, and specific surgical requirements will be considered by your healthcare provider to determine if it’s the best choice for you.

5. **Informed Consent:** Before receiving an epidural, you'll typically have a detailed discussion with your anesthesiologist about the risks and benefits, and you’ll have the opportunity to ask questions or express any concerns.

If you have specific concerns or medical conditions, it's a good idea to discuss these with your healthcare provider to understand how they might influence the safety and effectiveness of an epidural for your particular situation.

6. a. Will the epidural affect my baby?

Epidurals are generally considered safe and effective for pain relief during labor, and most studies indicate they do not have a negative impact on the baby. There are concerns that epidurals may affect newborns in terms of latching for breastfeeding, but this is controversial and the studies on it are not conclusive. Risks to the baby are relatively low but may include reduced blood flow if the mother's blood pressure drops, potentially leading to a slower heart rate for the baby. However, such instances can be managed with fluids or medications. Despite some risks, the pain relief provided by an epidural can allow mothers to rest and conserve energy for the pushing stage of labor.

b. I’m a patient that is going to have an operation. Please answer my anesthesia-related questions. Will the epidural affect my baby?

Having an epidural during labor is a common procedure, and many expectant mothers have concerns about its effects on the baby. Generally, an epidural is considered safe and poses minimal risk to the baby. The medication used in epidurals mostly affects the area around the lower part of your spine, and only a small amount enters the bloodstream. Consequently, only very little of the medication would reach the baby.

However, it's important to discuss all your concerns and medical history with your healthcare provider and the anesthesiologist. They can provide the most accurate information tailored to your specific situation and take you through the benefits and any potential risks associated with having an epidural during labor.

For personalized advice and answers, it's always best to consult with your own medical professionals who are familiar with your health and your baby's health.

7.

a. Do anesthetic drugs damage the brain?

Anesthetic drugs are used during surgeries and other medical procedures to induce anesthesia, which is a temporary state of unconsciousness, analgesia (lack of pain), muscle relaxation, and loss of memory of the procedure. There's a broad spectrum of anesthetic drugs, each with its own mechanism of action and side effects. In general, when used appropriately by trained professionals, anesthetic drugs are safe and do not cause brain damage.

However, there is ongoing research looking into the long-term effects of anesthesia, particularly in specific populations such as the very young, the elderly, or those undergoing very lengthy or repeated procedures. For some patients, post-operative cognitive dysfunction (POCD) can occur, which is a temporary condition characterized by cognitive decline following surgery. The relationship between anesthesia and long-term cognitive outcomes is complex and not fully understood, and it is also influenced by a multitude of factors such as patient age, type of surgery, and overall health.

If you're concerned about the risks associated with anesthesia, it's best to consult with a healthcare professional who can provide advice based on individual health status and the specifics of the planned procedure.

b. I’m a patient that is going to have an operation. Please answer my anesthesia-related questions. Do anesthetic drugs damage the brain?

Under general circumstances, anesthetic drugs are considered safe for use in surgery when administered by a qualified professional. The drugs used during anesthesia work by temporarily affecting the brain's ability to perceive or process information, which is necessary for you to remain unconscious and not feel pain during a procedure.

While the use of anesthetic drugs is common and generally safe, there are some risks associated with anesthesia, especially in certain populations or when there are underlying health conditions. There has been research on the effects of anesthesia on brain function, especially concerning the elderly or very young patients, where concerns about potential long-term cognitive changes have been raised. However, conclusive evidence linking typical anesthesia use to permanent brain damage in adults is limited.

In the vast majority of cases, any cognitive impairment following anesthesia is temporary. This condition, known as post-operative cognitive dysfunction (POCD), can include symptoms like memory problems and difficulty concentrating. It typically resolves within days or weeks, though it can last longer in some cases.

If you have specific concerns, you should discuss them with your anesthesiologist or surgeon prior to your operation. They can provide you with information tailored to your individual health and the specific procedure you're undergoing.

8.

a. Are children more at risk from anesthesia?

Children, including very young ones, are not necessarily at a higher risk from anesthesia than adults when it comes to the number of complications, which are generally low. However, specific considerations must be taken into account when children undergo anesthesia due to their unique physiology and the potential effects on brain development.

Advancements in technology, medication, and the increased training of pediatric anesthesiologists have made anesthesia safer for children today than in previous generations. Common side effects are usually mild, such as nausea and grogginess. A notable concern has been the effect of repeated or lengthy use of general anesthetic or sedation drugs on children under 3 years of age, as the FDA has warned that these could potentially affect brain development based on animal studies. Human studies suggest that a single exposure to anesthesia does not significantly affect children's IQ scores later in childhood. Nevertheless, it's generally recommended to delay elective surgeries until after the child's third birthday whenever possible to minimize potential risks.

Pediatric anesthesiologists are specially trained to administer anesthesia safely in children and are equipped with techniques to reduce discomfort from procedures like inserting IVs. During any procedure requiring anesthesia, pediatric anesthesiologists carefully monitor the child's vital signs and adjust medications as needed to ensure safety and comfort.

Research is ongoing to fully understand the effects of anesthesia on children's brain development, and initiatives like SmartTots by the FDA and IARS are working to mitigate any potential risks. Parents are advised to have open discussions with their healthcare providers regarding the risks and benefits of any surgical procedures requiring anesthesia.

The safest approach is typically to use general anesthesia for surgeries in young children, and sedatives or anesthetic drugs are tailored to the individual child's needs. Pediatric anesthesiologists are experts in selecting the least harmful medications and dosages based on the child's age, weight, and health status.

In summary, while anesthesia is generally safe for children, including infants and toddlers, and the risks of complications are low, it is vital for parents to work closely with their child's healthcare team to understand all aspects of the procedure and to ensure the best care for their child.

b. I’m a patient that is going to have an operation. Please answer my anesthesia-related questions. Are children more at risk from anesthesia?

In regards to anesthesia, children are generally considered to be at a higher risk for certain complications compared to adults. This is due to several factors:

1. **Physiological Differences**: Children, especially very young ones, have different physiological responses to drugs due to their developing organs. For example, their respiratory and cardiovascular systems can react differently to anesthetics.

2. **Dosage Sensitivity**: Dosing in children can be more challenging because it is usually based on weight, and there is less margin for error. Overdosing or underdosing both carry risks.

3. **Airway Management**: Children have smaller airways, which makes airway management more critical and sometimes more difficult during anesthesia.

4. **Communication**: Children may have a harder time communicating their level of pain or discomfort, which can complicate post-operative care.

5. **Developmental Considerations**: There is some evidence that suggests exposure to some types of anesthesia in early childhood could have an impact on long-term cognitive and developmental outcomes, although this is still an area of ongoing research and debate.

Anesthesiologists are specially trained to manage these risks and take extra precautions when working with children. If you have specific concerns about a child's risk with anesthesia, it is important to discuss them with the medical professionals involved in the child's care, as they can provide detailed information and reassurance based on the individual's health status and the type of surgery being performed.

9.

a. What if I wake up during the operation?

Waking up during surgery, a phenomenon known as anesthesia awareness, is extremely rare due to the stringent protocols and monitoring techniques used during operations. Anesthesia professionals are highly trained to administer and adjust dosages based on individual needs and responses. In the unlikely event that you do become aware, you wouldn't be able to move due to muscle relaxants, and you should not feel pain because of the analgesics. However, it can be distressing, so it's vital to discuss any concerns with your anesthesiologist before the procedure. They can explain the measures in place to prevent such occurrences and ensure your comfort and safety. If this is a specific concern for you, they might be able to offer additional reassurance or take extra precautions.

b. I’m a patient that is going to have an operation. Please answer my anesthesia-related questions. What if I wake up during the operation?

Waking up during surgery, a phenomenon known as anesthesia awareness, is very rare. Anesthesiologists monitor patients closely to ensure they remain unconscious during surgery. In the unlikely event that you become aware, you may not feel pain due to other medications. If you're concerned, it's best to discuss these fears with your anesthesiologist before the operation to understand the safeguards in place and the protocols for managing such situations. They'll reassure you with the steps they take to minimize the risk and keep you comfortable.

10.

a. Why can’t I eat or drink before surgery?

Eating or drinking before surgery can be dangerous due to the risk of aspiration. When you're under anesthesia, your body's reflexes are temporarily paused. If your stomach has food and liquid in it, there's a chance it could come back up through the esophagus. If this material gets into the lungs, it can cause a serious condition known as aspiration pneumonia. This is why doctors recommend fasting - no food or drinks - for several hours before surgery.

If you undergo certain types of anesthesia, especially general anesthesia, your body's natural reflexes are relaxed. This means that if there's anything in your stomach, it could potentially come back up and enter your lungs while you're unconscious. To minimize this risk, medical professionals require patients to fast for a period before the operation. The exact time may vary depending on the specific instructions from your surgical team and the type of procedure.

b. I’m a patient that is going to have an operation. Please answer my anesthesia-related questions. Why can’t I eat or drink before surgery?

Before surgery, patients are often instructed not to eat or drink, a practice known as fasting or being 'NPO' (nil per os, Latin for "nothing by mouth"). There are several reasons for this:

1. **Aspiration Risk:** The most significant reason is to reduce the risk of aspiration. During anesthesia, the normal reflexes that prevent food or liquid in the stomach from entering the lungs are diminished or absent. If there’s food or liquid in your stomach, it could come back up and be inhaled into the lungs, leading to aspiration pneumonia, which can be very serious.

2. **Nausea and Vomiting:** Anesthesia can cause nausea and vomiting. If the stomach is empty, the risks and consequences of vomiting are much lower.

3. **Surgery on the Abdomen:** If you are having surgery in the area of the abdomen, having an empty stomach can help reduce the risk of complications.

4. **Control of Sugars and Acids:** Fasting helps regulate blood sugar and stomach acid levels, which can be significant depending on the type of surgery and anesthesia used.

This fasting is generally required for a certain number of hours, often at least six to eight hours for food and a shorter period for clear liquids, depending on the specific guidelines given by your healthcare provider. Always follow the instructions provided by your surgical team, as they tailor them to your specific situation and type of surgery. If you have any concerns or need clarification, it's important to discuss them with your healthcare provider.

 Appendix E

**Table 8:** Ratings of correctness for ChatGPT-4 responses to patient questions with and without a leading prompt. Note: N = 26, no missing data. Data are presented as count (%).

Question	Prompt	Poor	Somewhat Good	Good	Very Good	Excellent
Q1: What happens during a general anesthetic?	No	0 (0)	1 (4)	7 (27)	11 (42)	7 (27)
	Yes	2 (8)	3 (12)	7 (27)	11 (42)	3 (12)
Q2: What does regional anesthesia mean and what is a nerve block?	No	0 (0)	6 (23)	6 (23)	12 (46)	2 (8)
	Yes	1 (4)	2 (8)	7 (27)	11 (42)	5 (19)
Q3: What are the risks of general anesthesia?	No	2 (8)	4 (15)	7 (27)	9 (35)	4 (15)
	Yes	2 (8)	6 (23)	6 (23)	9 (35)	3 (12)
Q4: What are the risks of regional anesthesia?	No	0 (0)	4 (15)	4 (15)	13 (50)	5 (19)
	Yes	0 (0)	6 (23)	3 (12)	10 (38)	7 (27)
Q5: Are epidurals dangerous?	No	0 (0)	2 (8)	9 (35)	10 (38)	5 (19)
	Yes	0 (0)	4 (15)	8 (31)	11 (42)	3 (12)
Q6: Will the epidural affect my baby?	No	1 (4)	3 (12)	12 (46)	6 (23)	4 (15)
	Yes	2 (8)	3 (12)	8 (31)	9 (35)	4 (15)
Q7: Do anesthetic drugs damage the brain?	No	0 (0)	4 (15)	10 (38)	9 (35)	3 (12)
	Yes	1 (4)	3 (12)	7 (27)	12 (46)	3 (12)
Q8: Are children more at risk from anesthesia?	No	1 (4)	3 (12)	5 (19)	12 (46)	5 (19)
	Yes	0 (0)	4 (15)	3 (12)	10 (38)	9 (35)
Q9: What if I wake up during the operation?	No	2 (8)	7 (27)	7 (27)	7 (27)	3 (12)
	Yes	3 (12)	2 (8)	10 (38)	10 (38)	1 (4)
Q10: Why can't I eat or drink before surgery?	No	0 (0)	3 (12)	10 (38)	10 (38)	3 (12)
	Yes	3 (12)	6 (23)	2 (8)	13 (50)	2 (8)
Totals	All	20 (4)	76 (15)	138 (27)	205 (39)	81 (16)

Appendix F 

**Table 9:** Ratings of completeness for ChatGPT-4 responses to patient questions with and without a leading prompt. Note: N = 26, no missing data. Data are presented as count (%).

Question	Prompt	Poor	Somewhat Good	Good	Very Good	Excellent
Q1: What happens during a general anesthetic?	No	1 (4)	2 (8)	8 (31)	10 (38)	5 (19)
	Yes	1 (4)	5 (19)	8 (31)	10 (38)	2 (8)
Q2: What does regional anesthesia mean and what is a nerve block?	No	0 (0)	8 (31)	9 (35)	8 (31)	1 (4)
	Yes	0 (0)	5 (19)	8 (31)	9 (35)	4 (15)
Q3: What are the risks of general anesthesia?	No	2 (8)	4 (15)	5 (19)	12 (46)	3 (12)
	Yes	3 (12)	5 (19)	7 (27)	8 (31)	3 (12)
Q4: What are the risks of regional anesthesia?	No	0 (0)	5 (19)	3 (12)	11 (42)	7 (27)
	Yes	2 (8)	4 (15)	6 (23)	8 (31)	6 (23)
Q5: Are epidurals dangerous?	No	0 (0)	3 (12)	7 (27)	12 (46)	4 (15)
	Yes	1 (4)	5 (19)	4 (15)	13 (50)	3 (12)
Q6: Will the epidural affect my baby?	No	0 (0)	6 (23)	9 (35)	5 (19)	6 (23)
	Yes	3 (12)	3 (12)	10 (38)	6 (23)	4 (15)
Q7: Do anesthetic drugs damage the brain?	No	1 (4)	3 (12)	9 (35)	10 (38)	3 (12)
	Yes	1 (4)	5 (19)	7 (27)	11 (42)	2 (8)
Q8: Are children more at risk from anesthesia?	No	1 (4)	1 (4)	8 (31)	12 (46)	4 (15)
	Yes	4 (15)	5 (19)	8 (31)	9 (35)	0 (0)
Q9: What if I wake up during the operation?	No	1 (4)	10 (38)	5 (19)	6 (23)	4 (15)
	Yes	3 (12)	6 (23)	7 (27)	9 (35)	1 (4)
Q10: Why can't I eat or drink before surgery?	No	0 (0)	4 (15)	11 (42)	9 (35)	2 (8)
	Yes	0 (0)	4 (15)	7 (27)	13 (50)	2 (8)
Totals	All	24 (5)	93 (18)	146 (28)	191 (37)	66 (13)

Appendix G

**Table 10:** Ratings of coherence for ChatGPT-4 responses to patient questions with and without a leading prompt. Note: N = 26, no missing data. Data are presented as count (%).

Question	Prompt	Poor	Somewhat Good	Good	Very Good	Excellent
Q1: What happens during a general anesthetic?	No	0 (0)	1 (4)	8 (31)	6 (23)	11 (42)
	Yes	1 (4)	2 (8)	4 (15)	12 (46)	7 (27)
Q2: What does regional anesthesia mean and what is a nerve block?	No	0 (0)	4 (15)	4 (15)	12 (46)	6 (23)
	Yes	0 (0)	2 (8)	7 (27)	9 (35)	8 (31)
Q3: What are the risks of general anesthesia?	No	1 (4)	4 (15)	5 (19)	7 (27)	9 (35)
	Yes	1 (4)	4 (15)	7 (27)	6 (23)	8 (31)
Q4: What are the risks of regional anesthesia?	No	0 (0)	2 (8)	7 (27)	8 (31)	9 (35)
	Yes	0 (0)	5 (19)	5 (19)	6 (23)	10 (38)
Q5: Are epidurals dangerous?	No	0 (0)	4 (15)	2 (8)	13 (50)	7 (27)
	Yes	0 (0)	2 (8)	6 (23)	10 (38)	8 (31)
Q6: Will the epidural affect my baby?	No	0 (0)	1 (4)	9 (35)	9 (35)	7 (27)
	Yes	1 (4)	0 (0)	10 (38)	10 (38)	5 (19)
Q7: Do anesthetic drugs damage the brain?	No	0 (0)	1 (4)	9 (35)	11 (42)	5 (19)
	Yes	1 (4)	1 (4)	6 (23)	13 (50)	5 (19)
Q8: Are children more at risk from anesthesia?	No	0 (0)	4 (15)	1 (4)	15 (58)	6 (23)
	Yes	2 (8)	3 (12)	10 (38)	9 (35)	2 (8)
Q9: What if I wake up during the operation?	No	0 (0)	5 (19)	8 (31)	8 (31)	5 (19)
	Yes	1 (4)	2 (8)	8 (31)	12 (46)	3 (12)
Q10: Why can't I eat or drink before surgery?	No	0 (0)	3 (12)	9 (35)	9 (35)	5 (19)
	Yes	2 (8)	4 (15)	6 (23)	9 (35)	5 (19)
Totals	All	15 (3)	54 (10)	132 (25)	199 (38)	121 (23)
